# Two Multicenter Surveys on Equine Back-Pain 10 Years a Part

**DOI:** 10.3389/fvets.2018.00195

**Published:** 2018-08-23

**Authors:** Barbara Riccio, Claudia Fraschetto, Justine Villanueva, Federica Cantatore, Andrea Bertuglia

**Affiliations:** ^1^Private Practitioner, Turin, Italy; ^2^Department of Veterinary Science, University of Turin, Grugliasco, Italy; ^3^Pool House Equine Clinic, Lichfield, United Kingdom

**Keywords:** back-pain, multicentric survey, sports medicine, veterinarians' opinion, equine spine

## Abstract

Despite back-pain being a common cause of poor performance in sport horses, a tailored diagnostic workflow and a consolidated therapeutic approach are currently lacking in equine medicine. The aim of the study was to assess the evolution in the veterinarian approach to diagnose and treat back-pain over a 10 years period. To investigate this topic, two surveys were addressed to equine veterinarians working in practice throughout Europe 10 years apart (2006 and 2016). The answers were organized in an Excel dataset and analyzed. There were 47 respondents in 2006 and 168 in 2016, from 8 European Countries. The main reasons for examining horses with back-pain were poor performance (76%), behavioral issues (68%), and lameness (50%). When assessing back pain, 97% of respondents applied careful digital pressure over paravertebral muscles, 90% of them used digital back mobilization, and 69% was detecting areas of localized heat. The use of diagnostic analgesia to confirm the source of pain was rarely employed. Radiography and ultrasonography were the most frequent diagnostic imaging modalities used to investigate the causes of back-pain in both surveys. Obtaining a definitive diagnosis in horses with back-pain is considered challenging due to the reduced accessibility of the area and the variability in the pain manifestations. Corticosteroids injections were used for local treatments by 80% of respondents in 2006 and 92% in 2016. Recently, ultrasonography has been extensively used during the injections of the vertebral articular facets and sacroiliac joints region. The use of complementary therapies was restricted to a low percentage of respondents in the first survey (20%) but it increased over the decade. In 2016, a wider percentage of respondents considered osteopathy (40%), kinesiotherapy (29%), and acupuncture (22%) when treating back disorders compared to 2006. The structural differences of the two surveys did not enable a direct data comparison. Based on the results of this surveys, however, veterinarians should be sensitized to the back-pain problems and seek to integrate findings from clinical research studies in their daily practice.

## Introduction

Back-pain is a common health problem in the equine population. It can cause chronic pain, limiting performance, and impair ability to work, which constitutes a common concern for veterinarians working with performance horses. Recently, the growing interest on equine back-pain has been demonstrated by the number of educational events have been organized and the increasing number of scientific manuscripts on the topic [1–4]. In absence of randomized clinical trials and systematic reviews of literature, there is still a perception of poor general consensus as to the clinical modalities and therapeutic routes to manage back-pain. Indeed, the authors have no knowledge of studies reporting current approach of veterinarians to back-pain and the exact influence of scientific researches on equine daily practice is not known. Therefore equine practices to treat back-pain could be determined more by empirical preference than scientific evidence, with marked geographical disparity.

The first aim of this study was to assess the current approach of practitioners to back-pain in horses. Therefore, a first survey conducted in 2006 and a second survey-based investigation realized in 2016 were analyzed. These two surveys resumed the general point of view of a cohort of veterinarians based in Europe on equine back-pain and the strategies to investigate and manage this condition. The second purpose of this work was to analyse the change of the clinicians' approach to equine back-pain over 10 years.

## Materials and methods

The Ethical Committee of the University of Turin approved the studies (Protocol Number 214499).

### Questionnaire composition

Two cross-sectional surveys were performed. The preliminary survey was conducted in 2006, containing 10 multiple-choice questions organized in a progressive order. The second survey was designed in 2016, sending a link for a web-based survey by email (Surveymonkey.com, Portland, Oregon). Additional questions were added in the 2016 questionnaire including 16 questions in total. In both cases, questions were translated into three different languages (English, French, and Italian) in order to reduce possible misunderstanding due to text interpretation.

In the first part of the survey, questions were focused on the veterinary surgeon's personal and professional information, including nationality, breed and use of the horses most commonly treated. The second questionnaire was anonymous and therefore it is impossible to know if responders to the first survey participated to the second one as well. Respondents' background was investigated to evaluate their experience as clinicians. Further questions were focused on diagnostic methods adopted and therapies preferred to treat horses with back-pain, including specific questions to evaluate the perceived efficacy of these diagnostic and therapeutic modalities. The questions related to the clinical examination, diagnostic techniques and treatment modalities required short answers such a simple “yes” or “no” and to indicate the frequency of use in case of positive answer. Otherwise, when the clinical value of specific behavior was investigated, a categorical scores system was employed with the following choices: no clinical value, poor clinical value, moderate clinical value, good clinical value, and excellent clinical value. The remaining questions required a multiple-choice closed answer; however free reply such as “other options” was always possible.

### Data collection and analysis

In the 2006 survey, invitations to participate to the study were posted directly by the principal investigators using a personal e-mail. The enrolment process was performed by direct selection of well-recognized equine specialists, working in referral veterinary hospitals in some European countries (France, Italy, United Kingdom, Suisse, Spain, Germany). For the 2016 survey, the potential target group required was wider. Therefore, the questionnaire was mailed to the secretary office of equine practitioners' national associations in France, Italy, Germany, United Kingdom, Ireland, Belgium, Denmark, Sweden and Suisse. A link referred directly to the online questionnaire was present in the e-mail. A cover letter with the investigator's contact information and the explanation of the study purposes was included. All equine veterinarians associated to their national society received the invitation to participate to this current study. If a secretary office of national association of equine practitioners was not present (Croatia), personal invitations were directly sent to equine practitioners working in referral centers in such countries. Incomplete responses, incomplete surveys and duplicate (surveys from veterinarians working in the same equine clinic/hospital) were excluded from the analysis. The answers were organized in an excel dataset and analyzed using a descriptive statistical method. Summaries and percentage were calculated by the survey programme.

## Results

Excluding the incomplete surveys, 47 responses were received in 2006; whilst in 2016, 168 respondents could be included in the study.

### Respondent's characteristics and study caseload

In the 2006 survey, complete replies were received from 6 European countries (14 each from France and Italy, 6 from both England and Sweden, 4 from Spain, and 3 from Germany). In 2016, a larger cohort of European nationalities participated to the study: the majority of the answers came from France (61 replies), Italy (26 replies), Germany (20 replies), Switzerland (9 replies), Belgium (23 replies), Spain (17 replies), Ireland (7), Denmark, and Croatia (2 replies for each of the last 2 countries). We received only 1 reply from England.

Participants to the 2006 study were all clinicians working in referral equine hospital, whereas participants of the 2016 survey were clinicians working in second opinion referral centers (55%), first opinion practitioners (36%) and equine specialists working in University Teaching Hospital (9%). The 11% of the responders of the 2016 study have been working in practice for <5 years, 16% for a period ≥5 and <10 years, 39% for ≥10 and <20 years and 34% for more than 20 years.

In 2006's survey, show-jumpers, dressage and eventers were classified as “competition horses” and this group was the most represented (53% of the equine population), 18% were Standardbreds, 15% Thoroughbreds, while pleasure horses were represented in a smaller proportion (8%), 2% of the horses' population were respectively Quarter horses, Spanish Riding Horses and horses used for Endurance. Instead in the 2016 survey, the equine sport disciplines more commonly served by respondents were represented by show-jumping, followed by pleasure riding, dressage, and ponies horse riding. Less frequently served equine sport disciplines were western riding, endurance, standardbred racehorses racing, eventing, thoroughbred racehorses racing, Polo, Driving, and Spanish riding (Table 1).

**Table 1:** Horses population characteristics examined by 2006 and 2016 survey respondents in clinical practice.

**Horses population (2016)**	**Draft**	**Dressage**	**Endurance**	**Eventing**	**Pleasure horses**	**Polo**	**Ponies**	**Quarter horses**	**Show-Jumpers**	**Spanish riding**	**STBRs**	***TBRs***
0–20%	163 (96)	112 (67)	139 (83)	148 (88)	91 (54)	163 (96)	129 (77)	134 (79)	52 (31)	163 (97)	139 (83)	146 (87)
20–50%	7 (4)	53 (31)	29 (17)	20 (12)	59 (35)	7 (4)	35 (21)	29 (18)	86 (51)	0 (0)	26 (15)	17 (10)
50–100%	0 (0)	3 (2)	0 (0)	0 (0)	18 (11)	0 (0)	3 (2)	5 (3)	30 (18)	5 (3)	3 (2)	5 (3)
**Horses population (2006)**	**Competition horses (Dressage, Show-Jumpers, Eventing)**	**Endurance**	**Pleasure horses**	**Quarter horses**	**Spanish riding**	**STBRs**	**TBRs**					
Number (%)	25 (53)	1 (2)	4 (8)	1 (2)	1 (2)	8 (18)	7 (15)					

*STBRs, Standardbred racehorses; TBRs, Thoroughbred racehorses*.

In 2006, 42% of the interviewed veterinarians stated to perform 50–100 musculoskeletal investigations per month. In 2016, the average number of horses examined for orthopedics problems per months was reduced, in fact only 5% of respondents perform more than 50 investigation and the majority (40%) of the responders examined less than 20 horses per month. In both studies, back-disorder was recognized just in a minority of the orthopaedic cases, with a value between 0 and 20% of the examined cases by the 70% of respondents.

### Clinical tests to detect back-pain in horses

The digital pressure of paravertebral muscles and the behavioral response to back mobilization tests resulted the two most commonly tests to detect back-pain in horses adopted by veterinarians in both surveys (the above-mentioned tests were always performed respectively by 98–85% of respondents in 2006, and by 97–90% of respondents in 2016). Less commonly, palpation was employed in detecting local heat over the back (by 60% of 2006's survey respondents and by 69% of respondents in 2016). The numbers of tests routinely used by the survey's respondents in 2006 was greater than in 2016 (Table 2). In particular, 78% of respondents commonly used the surcingle test in 2006, however 38% of them did not employ this method in 2016. Diagnostic analgesia was occasionally performed during back work-up by the totality of the responders in 2006, but 38% of them did not include this procedure during back evaluation in 2016. The digital evaluation of local thickening of the supraspinous ligament and the ridden evaluation (always performed by the 65% and the 82% of respondents in the 2006 survey) were only occasionally performed in 2016 (by the 38% and the 70% of veterinarians).

**Table 2:** Clinical tests used by the 2006 and 2016 survey respondents in order to detect back-pain in horses.

**Clinical tests**	**2016 Respondents** ***n*** **(%)**			**2006 Respondents** ***n*** **(%)**		
	**Always**	**Sometimes used**	**Never used**	**Always**	**Sometimes used**	**Never used**
Back mobilization	**151 (90)**	12 (7)	5 (3)	**40 (85)**	7 (15)	0 (0)
Diagnostic analgesia	8 (5)	57 (34)	**102 (61)**	0 (0)	**47 (100)**	0 (0)
Evaluation of saddle	**76 (45)**	**82 (49**)	10 (6)	n.r.	n.r.	n.r.
Local heat areas	**116 (69)**	25 (15)	25 (15)	**28 (60)**	13 (27)	6 (13)
Local thickening of supraspinous ligament	40 (24)	**64 (38)**	**64 (38)**	**31 (65)**	12 (26)	4 (9)
Neurological examination	32 (19)	**118 (70)**	18 (11)	0 (0)	**47 (100)**	0 (0)
Oral examination	52 (31)	**89 (53)**	27 (16)	n.r.	n.r.	n.r.
Paraspinal muscles digital pressure	**163 (97)**	2 (1)	3 (2)	**46 (98)**	1 (2)	0 (0)
Rectal examination	13 (8)	**101 (60)**	54 (32)	10 (21)	**31 (66)**	6 (13)
Ridden exercise evaluation	10 (6)	**118 (70)**	40 (24)	**39 (82)**	8 (18)	0 (0)
Surcingle test	34 (20)	**71 (42)**	**64 (38)**	**41 (87)**	6 (13)	0 (0)

*n.r., not required. Frequently reported percentage (>35%) are given on bold font*.

Nevertheless, the range of the diagnostic tests performed in 2016 was extremely variable including rectal examination, oral examination, evaluation of the saddle's fit, and neurological examination. In particular, this latter showed an increase in its use compared to the 2006 survey. An osteopathic evaluation was mentioned multiple times as a part of the evaluation routinely performed to detect back-pain among the open answers given by respondents in 2016.

The clinical value attributed to the commonest clinical modalities was investigated in the 2016 survey. Physical examination of the thoracolumbar spine using the mobilization tests or the digital pressure over the epaxial muscles was considered as “excellent” or “good” method to evaluate the back region according to 89 and 81% of the respondents, respectively. The detection of areas of local heat was considered having “good” to “moderate” clinical value for 33 and 29% of the respondents. According to 41% of the respondents, the rectal palpation of the pelvis had low sensitivity in detecting problems at the level of the axial skeleton. The clinical value given to diagnostic analgesia was contradictory: 27% of respondents considered it as reliable, whereas the 23% of respondents assigned no clinical value to it. All the other diagnostic tests were comprehensively considered having “low” to “moderate” value.

### Signs suggestive of back-pain

According to 2006's survey, clinical signs suggestive of back-pain in horses were unwillingness to work (96%), modification of the jumping technique and poor performance (89%), loss in gait amplitude (85%), and reluctance to turn during ridden exercise (81%). Less commonly, respondents mentioned subtle hindlimbs lameness (55%), modification in the trajectory during ridden exercise (53%), the presence of areas of focal heat (40%), and forelimbs lameness (21%). The presenting complaints for horses suffering from back-pain for the 2016 respondents were poor performances (76%) and non-specific problems (68%), such as behavioral issues, reluctance to jump, or difficulties with the farrier and lameness (50%). Paravertebral muscle atrophy, difficulty riding the horse, resistance, or difficulty in transition during ridden exercise, reluctance to jump, obvious discomfort and spasm of *longissimus dorsi* at palpation were also described. A bunny-hopping gait or the exhibition of bad attitude during work were considered clinical signs suggestive of pain at this level (Table 3).

**Table 3:** Clinical signs considered by respondents in 2006 and 2016 suggestive of back-pain in horses.

**Clinical signs**	**2016 Respondents *n* (%)**	**2006 Respondents *n* (%)**
Aggressive behavior	134 (81)	n.r.
Bad attitude	102 (61)	n.r.
Bunny-hopping hindlimb gait	94 (56)	n.r
Difficulty during transition	121 (72)	n.r
Difficulty to curve	116 (69)	38 (81)
Difficulty to ride/Resists work	131 (78)	45 (96)
Drifting away during work	92 (55)	25 (53)
Local heat area	74 (44)	19 (40)
Loss of amplitude in the gaits	129 (77)	40 (85)
Modification of jumping style	138 (82)	42 (89)
Paravertebral muscle atrophy	133 (79)	n.r.
Poor hindlimbs impulsion	119 (71)	n.r.
Poor performances	124 (74)	42 (89)
Refuse to jump	113 (67)	n.r.
Spasm of longissimus dorsi at palpation	111 (66)	n.r.
Subtle hindlimb lameness	82 (49)	26 (55)
Unexplained forelimb lameness	67 (40)	10 (21)

*n.r., not required*.

### Diagnostic imaging techniques

Results of both studies were similar, confirming that radiography and ultrasonography were the preferred modalities to image the axial skeleton.

In 2016, 45% of the respondents declared to use radiography during back work-up while 50% just occasionally. Ultrasonography was always included in the spinal evaluation by 25% of the clinicians while 70% of them used it occasionally. The majority of the respondents (70%) considered radiography “good” or “excellent”, and 40% of respondents rated ultrasonography as a “good” technique. The use of scintigraphy was limited (only the 2% of respondents) although its clinical value was considered “excellent” according to 23% of respondents and “good” according to 41% of the responders. Thermography was rarely employed in clinical setting (88% of respondents has never used it) and 30% of respondents considered it as unreliable (Table 4).

**Table 4:** Frequencies of imaging modalities used to diagnose spinal pathologies by 2006 and 2016 survey respondents.

**Imaging modalities**	**2016 Respondents** ***n*** **(%)**			**2006 Respondents** ***n*** **(%)**		
	**Always**	**Sometimes used**	**Never used**	**Always**	**Sometimes used**	**Never used**
Radiology	**76 (45)**	**84 (50)**	8 (5)	**23 (49)**	**22 (47)**	7 (4)
Scintigraphy	3 (2)	**97 (58)**	**67 (40)**	15 (9)	**25 (53)**	**18 (38)**
Thermography	2 (1)	18 (11)	**148 (88)**	3 (2)	9 (19)	**37 (79)**
Ultrasonography	40 (24)	**118 (70)**	10 (6)	10 (21)	**30 (64)**	25 (15)

*Frequently reported percentage (>35%) are given on bold font*.

Although these techniques were suitable to diagnose back-pain syndrome, veterinarians were rarely able to identify a primary pathology in this site. Primary back problems were encountered in 50% of the cases in 2006 and in 46% in 2016; on the other hand back-pain syndrome was secondary to lameness in 49% of the cases in 2006 and 54% in 2016. The pathologies more commonly detected in 60–80% of the cases suffering from primary back-pain according with 2016 survey were kissing spine (15%) and osteoarthritis of the thoracolumbar articular facets (14%) (Table 5).

**Table 5:** Primary back pathologies and corresponding frequencies identified in the case-load of horses with back-pain by 2016 survey respondents.

**Primary back pathologies**	**0–10% of cases *n* (%)**	**10–20% of cases *n* (%)**	**20–40% of cases *n* (%)**	**40–60% of cases *n* (%)**	**60–80% of cases *n* (%)**	**>80% of cases *n* (%)**
Kissing spine	7 (4)	44 (26)	49 (29)	42 (25)	25 (15)	2 (1)
Muscle strains	**83 (49)**	34 (20)	25 (15)	12 (7)	8 (5)	7 (4)
OA of the TL articular facets	29 (18)	34 (20)	57 (34)	22 (13)	24 (14)	2 (1)
Sacroiliac DJD	22 (13)	**74 (44)**	37 (22)	22 (13)	12 (7)	2 (1)
Sacroiliac ligament desmitis	32 (19)	**70 (41)**	27 (16)	27 (16)	13 (8)	0 (0)
Stress fractures back/pelvis	**139 (83)**	20 (12)	7 (4)	2 (1)	0 (0)	0 (0)
SL desmitis	47 (28)	**82 (49)**	23 (14)	10 (6)	3 (2)	2 (1)
Ventral spondylosis	**109 (65)**	40 (24)	15 (9)	2 (1)	2 (1)	0 (0)

*OA, osteoarthritis; DJD, degenerative joint disease; TL, thoracolumbar; SL, supraspinous ligament. Frequently reported percentage (>35%) are given on bold font*.

### Therapeutic modalities to treat back-pain

In 2006 survey respondents had a favorable perception of intramuscular and intravenous drugs administrations (according to 55% of respondents). The ultrasound-guided (US-guided) techniques for administration of drugs in the sacroiliac region was judged fairly uncertain by 43% of respondents in 2006, and 40% of respondents had the same opinion for the US-guided medication performed at the level of the thoracolumbar articular facets and for mesotherapy. Likewise, the 32% of respondents ignored the effect of paravertebral injections.

In contrast, in the 2016 survey the systemic administration of drugs was employed routinely only in 2% of cases. The treatments commonly employed were mesotherapy, US-guided injection of thoracolumbar facets, the US-guided injection of the sacroiliac joint region and the injection of the dorsal spinous processes interspace (Table 6). During the considered decade, thoracolumbar facets injection and sacroiliac region injection under US-guidance were perceived having a superior efficacy compared to the same techniques performed blindly, and mesotherapy has been perceived to be effective by a large number of respondents (Table 7).

**Table 6:** Therapeutic routes and corresponding frequencies to treat back disorders in horses by 2016 survey respondents.

**Therapeutic modalities for drugs administration to treat back-pain in 2016 survey**	**0–10% of cases *n* (%)**	**10–20% of cases *n* (%)**	**20–40% of cases *n* (%)**	**40–60% of cases *n* (%)**	**60–80% of cases *n* (%)**	**80–90% of cases *n* (%)**	**90–100% of cases *n* (%)**
IM or IV route	**70 (41)**	45 (27)	32 (19)	10 (6)	7 (4)	2 (1)	3 (2)
Medication between spinous processes	29 (17)	40 (24)	45 (27)	20 (12)	7 (4)	17 (10)	10 (6)
Mesotherapy	39 (23)	24 (14)	18 (11)	20 (12)	25 (15)	27 (16)	15 (9)
Paravertebral injection	30 (18)	42 (25)	30 (18)	34 (20)	15 (9)	15 (9)	2 (1)
Sacro-iliac joint injection	**84 (50)**	37 (22)	17 (10)	13 (8)	8 (5)	5 (3)	3 (2)
US-guided medication of the TL articular facets	**66 (39)**	22 (13)	25 (15)	20 (12)	8 (5)	15 (9)	12 (7)
US-guided sacro-iliac joint medication	52 (31)	34 (20)	27 (16)	25 (15)	7 (4)	13 (8)	10 (6)

*IM, intramuscular; IV, intravenous; TL, thoracolumbar. Frequently reported percentage (>35%) are given in bold font*.

**Table 7:** Perceived efficacy of different therapeutic modalities to treat back-pain in horses by 2006 and 2016 survey respondents.

**Perceived efficacy of therapeutic modalities in 2016**		**None *n* (%)**	**Poor *n* (%)**	**Moderate *n* (%)**	**Good *n* (%)**	**Excellent *n* (%)**
General administration of NSAIDs		39 (23)	**57 (34)**	**54 (32)**	17 (10)	2 (1)
General administration of tiludronate		49 (29)	34 (20)	**59 (35)**	84 (15)	2 (1)
General administration of steroids		30 (18)	**66 (39)**	**62 (37)**	10 (6)	0 (0)
IRAP		**79 (47)**	40 (24)	34 (20)	15 (9)	0 (0)
Medication between spinous process		12 (7)	27 (16)	49 (29)	**62 (37)**	17 (10)
Medication of sacro-iliac joint		38 (14)	34 (20)	44 (26)	**59 (35)**	8 (5)
Paravertebral medication of the TL articular facets		10 (6)	29 (17)	40 (24)	**60 (36)**	44 (16)
Paravertebral US-guided medication of the TL articular facets		5 (3)	10 (6)	32 (19)	**100 (59)**	20 (12)
US-guided medication of sacro-iliac joint		5 (3)	12 (7)	32 (19)	**97 (58)**	22 (13)
Mesotherapy		29 (17)	27 (16)	42 (25)	**54 (32)**	17 (10)
PRP		**92 (55)**	84 (15)	35 (21)	15 (9)	0 (0)
**Perceived efficacy of therapeutic modalities in 2006**	**Don't know** ***n*** **(%)**	**Poor response** ***n*** **(%)**	**Moderate response** ***n*** **(%)**	**Good response** ***n*** **(%)**	**Excellent response** ***n*** **(%)**	**Inconstant response** ***n*** **(%)**
IM or IV route	3 (6)	2 (4)	13 (27)	**26 (55)**	2 (4)	2 (4)
Mesotherapy	**19 (40)**	3 (6)	4 (9)	15 (32)	4 (9)	2 (4)
Paravertebral injection	30 (18)	42 (25)	30 (18)	34 (20)	15 (9)	15 (9)
Sacro-iliac joint medication	**20 (43)**	4 (8)	3 (7)	14 (30)	2 (4)	4 (8)
US-guided paravertebral medication	**19 (40)**	1 (2)	1 (2)	12 (25)	13 (28)	1 (3)
US-guided sacro-iliac joint medication	**23 (49)**	2 (4)	2 (4)	11 (23)	22 (17)	1 (3)

*NSAIDs, non-steroids anti-inflammatory drugs; IRAP, interleukin-1 receptor antagonist protein; US, ultrasound; PRP, platelets rich plasma; TL, thoracolumbar. Frequently reported percentage (>35%) are given in bold font*.

*IM, intramuscular; IV, intravenous; US, ultrasound. Frequently reported percentage (>35%) are given in bold font*.

### Drugs and therapeutic preparations

In both survey this section was divided in two parts consisting in drugs administered locally or systemically (Table 8). Corticosteroids were the drugs more commonly used among respondents for local treatment, followed by distillate of Sarracenia Purpurin, while anti-inflammatory non-steroid drugs (NSAIDS) were the commonest drugs administered using the general route (49%).

**Table 8:** Classes of drugs administered to treat back-pain by 2006 and 2016 survey respondents.

**Classes of drugs**	**2016**		**2006**	
	**General *n* (%)**	**Local *n* (%)**	**General *n* (%)**	**Local *n* (%)**
Biological therapies (IRAP, PRP)	0 (0)	15 (9)	0 (0)	0 (0)
Local anesthetic	0 (0)	**84 (50)**	0 (0)	13 (28)
Central muscle relaxants	0 (0)	34 (20)	6 (13)	7 (15)
NSAIDs	40 (24)	0 (0)	**23 (49)**	0 (0)
Homeopathic	0 (0)	27 (16)	3 (7)	6 (13)
Sarracenia purpurin	0 (0)	**64 (38)**	1 (2)	**22 (47)**
Steroids	17 (10)	**138 (82)**	3 (6)	**38 (80)**
Tiludronate	34 (20)	0 (0)	3 (6)	0 (0)
Others	0 (0)	13 (8)	2 (4)	4 (8)

*IRAP, interleukin-1 receptor antagonist protein; PRP, platelets rich plasma; NSAIDs, non-steroids anti-inflammatory drugs. Frequently reported percentage (>35%) are given in bold font*.

Respondents confirmed that local injection of corticosteroids was their first therapeutic choice treating back-pain, with a predilection for dexamethasone. Different molecules have been employed during the analyzed decade, in particularly local anesthetic drugs such lidocaine was frequently employed for mesotherapy (50% of respondents), Sarracenia purpurin (38% of respondents) for local analgesia, and bisphosphonates (19%). The NSAIDS were used less frequently in 2016 than in 2006 (24% of respondents), whereas 20% of respondents indicated to use central muscles relaxants, such tiocolchicoside and metocarbamol for general and for loco-regional route. There was no significant increase in the use of homeopathies (like Traumeel® and Zeel® injected locally), employed by the 13% of respondents in 2006 and by the 16% in 2016. The local use of vitamin-B and other preparations such as Interleukin 1-Receptor Antagonist Protein (IRAP®), Platelet-Rich Plasma (PRP), Hyaluronic Acid, sodium chloride 0.9%, Iodine, and Ozone were mentioned but not frequently used.

Based on the results of 2016 survey, the general perception is that drugs are more effective if administered locally rather than via the general route. Interestingly, the perceived therapeutic efficacy of corticosteroids, NSAIDS and bisphosphonates administered via general route was “poor” to “moderate” for 76, 66, and 55% of the respondents. Similarly, the perceived efficacy attributed to IRAP and PRP was “none” or “poor” according to 71 and 70% of respondents, respectively.

### Complementary therapies

**Figure 1:**
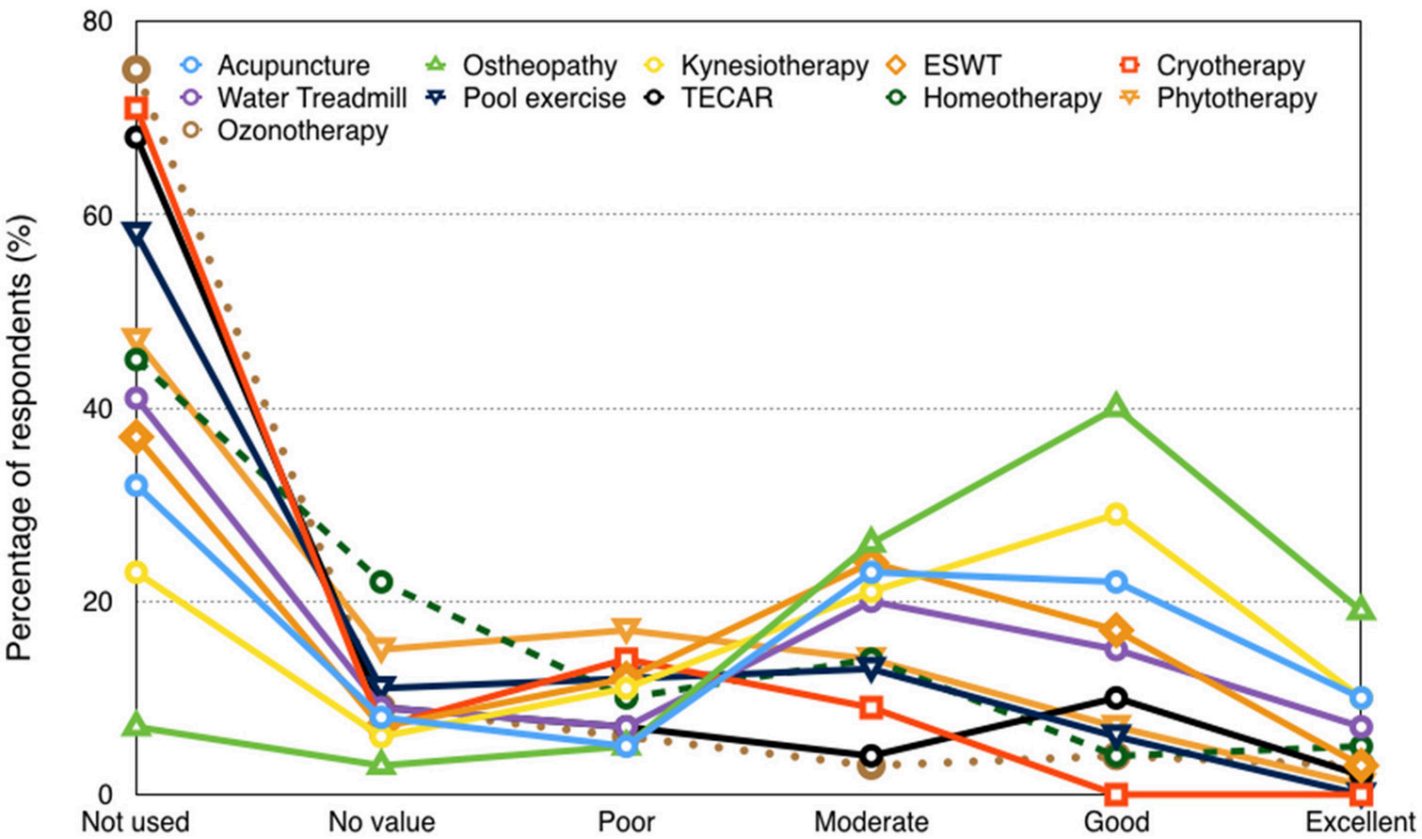
Efficacy of the complementary therapies in treating horse's back disorders.

In both 2006 and in 2016 surveys, a low percentage of respondents (<20%) prescribed complementary therapies to treat back-pain, however the use and efficacy perception of osteopathy were significantly increased during the last decade (Figure 1). The percentage of respondents considering osteopathy as an “excellent” technique treating back disorders increased from 0% in 2006 to 40% in 2016. Kinesiotherapy was considered “good' or “excellent” in a high proportion of responders (39%) as well. In the 2016 survey, the majority of respondents stated not to use the following therapeutic modalities: cryotherapy (71%), ozone therapy (75%), capacitive-resistive diathermy (68%), homeotherapy (45%), and phytotherapy (47%). Although extracorporeal shock waves therapy has never been used by the 37% of respondents, a considerable proportion (20%) considered it as “good” or “excellent.” Similarly, acupuncture has never been employed by the 32% of respondents, but a large percentage of respondents considered its efficacy “good” or “excellent.” Laser phototherapy has never been used by nearly half of the interviewed veterinarians (48%), however 12% of them considered it “good” or “excellent.” Although water-treadmill has never been employed by the 41% of respondents for rehabilitative purposes, the general perception was positive by 52% of respondents. Finally, the 58% of respondents stated not to advise to use swimming pool for rehabilitation of back problems.

## Discussion

The current questionnaires are the first international surveys on back-pain management in sport horses. Two different multicentre surveys have been employed to collect veterinarians' opinions on equine back-pain syndrome over the last decade in a limited number of European countries. The restricted number of respondents from few Europeans countries does not allow giving a generalized portrait of the European trends in veterinary practice. Excellent feedback was obtained from French veterinarians in 2006 and in 2016, which likely created some degree of bias. Although just few countries were included in the investigations, the study is highly informative in light of the number of veterinary surgeons participating to it.

The major limitation is that the two surveys have been addressed to different groups of veterinarians, sampling different population of horses. Respondents represented a varied group of veterinarians with different experience, working in different clinical setting. It would have been interesting to restrict the interview to the same respondents to the previous survey to define the evolution in their approach over 10 years. However, the authors felt it would have been more appropriate to increase the number and the variability of the veterinary surgeons participating at the second study, including also veterinarians working in first opinion practices. Due to this major limitation, the second aim of the study was not achieved. Furthermore, in 2016 the casework of the interviewed veterinarians included a lower number of lameness investigations compared to 2006, when the majority of the enrolled clinicians were performing 50–100 investigation a month. This reflects a different caseload of horses sampled in our study; partly it could be the effect of the diffuse economic recession or the results of the sampling technique. Although a random method of selection of the study sample would have been more appropriate, it was impossible to select our sample in such way. The equine population considered in the surveys was not represented by a heterogeneous group of horses, with an highest prevalence for sport horses competing in equestrian disciplines and for a population of hospital attenders' competition horses; therefore, the results of the study cannot be generalized to the whole equine population.

Axial skeletal problems are one of most common injuries in horses performing equestrian disciplines [5, 6]. The estimated prevalence of back problems in literature varies from 0.9 to 94% of the ridden horse population [7], depending on type and level of activity. The breed of horses served by the veterinary practice and the expertise of the operator evaluating back-pain could have influenced the extreme variability in this range, as demonstrated by a previous studies [5, 8, 9]. Training intensity and the specific sport discipline may increase the risk of such specific injuries [5, 6, 10]; however, the present study has not the purpose of drawing conclusions on the prevalence of back-pain syndrome in different equestrian specialties.

As reported in literature and confirmed in the current study, clinical signs of back-pain are various and poor specific [1–4, 8, 11]. Usually clients of horses affected by the syndrome reported a reduction in performance and behavioral issues. Interestingly, this data reflects an increase attention to the horse's ethogram by the clients, in accordance with the current veterinary literature [12, 13]. Respondents reported aggressive response to back manipulation and difficulty during ridden exercise/reluctance to work as the main hallmarks of back-pain in horse especially during ridden exercise. Nowadays, changes in behavior are considered one of the main manifestations of back-pain by equine specialists [1–4, 8, 9, 14]. In spite of this fact, the veterinarians are still reluctant to correlate clinical signs of back-pain with a primary spinal pathology and back disorders were considered as main source of pain only in the minority of cases. The association between lameness and back problems in horses is frequently discussed among equine practitioners [1–4, 9, 14–18]. Chronic subclinical lameness may have an impact on spinal biomechanics and kinematics [1–4, 14–16], and on the other hand lameness could be secondary to spinal dysfunction [14, 15]. In the 2016 survey, our respondents reported that lameness was observed in <50% of the patients suffering from back-pain.

Atrophy of the paravertebral muscles is consistently related to back-pain in the opinion of veterinarians (according to 79% of respondents to the last survey) since it reflects a reduced function, providing information on the presence of pain and underlying lesions [19]. The role of epaxial muscles in the spinal stabilization and stiffen has been analyzed in several studies in the last decade [2, 3, 20, 21] and secondary atrophy of *longissimus dorsi* and *multifidus* muscles has been described in horses suffering from pain localized to thoracolumbar region [9, 20, 22, 23]. Therefore, in respondents' experience, the evaluation of the muscular system could be highly suggestive of spinal pathology. For the same reason, the subjective evaluation of the back flexibility and the subjective evaluation of animal response to digital pressure over the paraspinal muscles remained the clinical tests more commonly performed in practice, perceived as highly useful by veterinarians. However, the assessment of the response to these tests to date is still based on the subjective evaluation rather than on objectively algometric data, even in presence of multiple studies reporting the effectiveness of mechanical nociceptive thresholds [17, 24]. The number of clinical tests used in 2016 was lower compared with 2006, suggesting that the veterinarians have selected more specific methods to detect back-pain over or that less time is dedicated to the static evaluation of the horse. The “surcingle test” and the diagnostic analgesia are currently rarely employed in practice. The “surcingle test” could be dangerous for both the horse and the operator in presence of severe back-pain (J. M. Denoix, personal communication) while diagnostic analgesia of the back has been previously criticized because the infiltration of local anesthetic could affect the spinal function even in clinically sound horses [25]. On the other hand, clinicians are routinely employing several different methods for detecting back disorders, even though the clinical value attributed to them is poor to moderate.

Concerning diagnostic modalities, over the last decade radiography and ultrasonography became more popular investigating the back region and the general perception is that they are highly effective diagnostic methods. Radiography is considered useful in detecting osteoarticular lesions in the thoracolumbar region [3, 22] but it has limitations due to the superimposition of the pelvis [3, 23]. For further investigation of the lumbar and pelvic region scintigraphy could be required [3, 22, 26]. Nevertheless, its use is still limited due to financial constraints. Ultrasonography is routinely employed by practitioners in the diagnosis and treatment of back disorders [27–29], however in both surveys the employment of ultrasonography was lower than radiography. In first instance, veterinarians are probably accustomed to use radiography more often than ultrasonography to identify back lesions and, as consequence, they have possibly still less experience in the interpretation of ultrasound images compared with radiographs. Our data show that thermography is not routinely employed in equine practice, even if one clinical study reported that is a not invasive and auxiliary method to identify lesions in the thoracolumbar region [30]. This result could be justified by the high variability of measurements due to the influence of environmental conditions [3, 31].

Although several therapeutic modalities are available, depending on the primary pathology and its severity [3, 19, 32–35], little objective information is present in literature on the current usage of different therapeutic modalities within equine practice. From the results of the last survey, it is possible to conclude that the veterinarians participating in our study preferred local medications, in contrast with what emerged in the previous questionnaire. Conclusions cannot be drawn due to the strong limitations of the study. However, the increased awareness of the advantages of local medications, together with the diffusion of US-guided techniques could explain this difference. In 2006, 40% of the respondents declared not to know the justification of US-guided injection of the sacroiliac region, facets joints and dorsal spinous processes whereas the majority of respondents routinely performed these procedures in 2016. The numerous studies evaluating the accuracy of US-guided injections in the axial and sacroiliac region compared to “blind techniques” could have helped the diffusion in clinical settings [34, 36–38]. Although there is limited evidence of its effectiveness [32, 33], mesotherapy was perceived as a therapy with good efficacy between respondents to our last survey. The topical administration was the prevailing route for drugs administration in the 2016' survey because perceived as more effective. The two surveys confirmed that corticosteroids are the main drug family used by the interviewed veterinarians to treat back disorders. Interestingly, the use of a distillate of powdered of pitchered plant (Sarracenia purpurin) as an analgesic agent is still widespread, although its efficacy with regard to horses is not documented in literature [33]. This data is surprising also considering the limited availability of the corresponding commercial preparations (Sarapin®, P-Block®) in most European countries and the counterproductive effect that could have in mesotherapy [32]. Instead, the use of systemic NSAID in the treatment of spinal pathologies seemed to decrease over the last decade because the limited clinical value encountered in comparison to the 2006' questionnaire. Interestingly, the use of systemic bisphosphonates has tripled comparing data between our two questionnaires, even if the majority of veterinarians considered them of limited clinical value. Controlled clinical trials have been published on bisphosphonates' effect in back-pain over the last 10 years [33, 39] and this could have influenced the use by practitioners during such period. The results of our study can indicate a limited tendency to use biological therapies, homeopathies (Traumeel®, Zeel®), central muscle relaxant, and other preparations locally injected (Sodium Clorure, iodine, or ozone) by European horse clinicians. Further researches such as clinical trials are necessary to justify their use in horses before thinking a considerable diffusion in practice. Manual therapies have been applied to horses treating musculoskeletal diseases [7, 35, 40, 41]. Osteopathic manipulations [21], kinesiotherapy and acupuncture [42] are perceived as good auxiliary treatments by our respondents. Despite the efficacy of extracorporeal shock wave therapy relieving deep muscular pain at the level of the back has been demonstrated [43] our study suggests its perceived efficacy by equine clinician is still moderate. A similar trend is registered for diathermy and ozonotherapy. The present study did not report surgical management of dorsal spinous processes impingement [44–47] despite three different studies from United Kingdom described encouraging results and therefore it effectiveness should perhaps be investigated in future [47–49]. The limited number of respondents by United Kingdom could be the main reason of this discrepancy between our data and the literature.

In conclusion, the present study gives an insight into the current perception of different clinicians working in different settings regarding horse back-pain, but it was not able to highlights the change in the veterinarians approach in the diagnosis and management of this condition over the last decade. Equine practitioners are conscious of the limitations related to the clinical tests and imaging techniques available for detecting back disorders. Achieving the correct diagnosis is still challenging, because of the restricted accessibility of this area and the variability of the pain manifestations. As a consequence, the advised treatment is often empirical and focus to improve the comfort of the horse instead of treating the origin of the problem. A multimodal approach is often required to manage this condition. In the absence of an objective method to assess pain in practice and consolidated protocols to treat back-pain problems, this study could be considered just as a starting point. Futures studies should be designed in order to rigorously collect follow-up from veterinarians in order to verify whether the common perception on several treatments is actually confirmed in clinical setting. The value gained interviewing the treating veterinarians instead of the owners is that the physicians should be able to assess the improvement more objectively, without being influenced by the client satisfaction.

## Author contributions

BR and AB conceived and designed the study. JV and AB contributed in an equal manner preparing surveys and to the acquisition of data. AB, BR, FC, and CF contributed to the interpretation of data. CF drafts the manuscript. BR, AB, and FC draft the paper and revising it critically for intellectual content.

### Conflict of interest statement

The authors declare that the research was conducted in the absence of any commercial or financial relationships that could be construed as a potential conflict of interest.
